# A Method for SUMO Modification of Proteins *in vitro*

**DOI:** 10.21769/BioProtoc.3033

**Published:** 2018-10-05

**Authors:** Christine C. Lee, Bing Li, Hongtao Yu, Michael J. Matunis

**Affiliations:** 1 Department of Biochemistry & Molecular Biology, Johns Hopkins Bloomberg School of Public Health, Baltimore, MD, USA; 2 Department of Pharmacology, Howard Hughes Medical Institute, University of Texas Southwestern Medical Center, Dallas, TX, USA

**Keywords:** SUMO, *In vitro* sumoylation assay, SUMO1, E1 activating enzyme, E2 conjugating enzyme

## Abstract

The Small Ubiquitin-related Modifier (SUMO) is a protein that is post-translationally added to and reversibly removed from other proteins in eukaryotic cells. SUMO and enzymes of the SUMO pathway are well conserved from yeast to humans and SUMO modification regulates a variety of essential cellular processes including transcription, chromatin remodeling, DNA damage repair, and cell cycle progression. One of the challenges in studying SUMO modification *in vivo* is the relatively low steady-state level of a SUMO-modified protein due in part to the activity of SUMO deconjugating enzymes known as SUMO Isopeptidases or SENPs. Fortunately, the use of recombinant SUMO enzymes makes it possible to study SUMO modification *in vitro*. Here, we describe a sensitive method for detecting SUMO modification of target human proteins using an *in vitro* transcription and translation system derived from rabbit reticulocyte and radiolabeled amino acids.

## [Background]

Like other ubiquitin protein family modifications, SUMO modification occurs through an ATP-dependent enzymatic cascade involving the sequential activity of an E1 activating enzyme (the Aos1/Uba2 heterodimer in humans), an E2 conjugating enzyme (Ubc9), and one of many E3 ligating enzymes (Gareau and Lima, 2010). Proteins with a SUMO conjugation consensus site, ΨKxE (Ψ is a hydrophobic residue, followed by a lysine, any amino acid, and glutamic acid), can be efficiently modified by one or several of the SUMO paralogs expressed in mammals, including SUMO1, SUMO2 or SUMO3 (collectively referred to as SUMO2/3, due to their 97% sequence homology) (Gareau and Lima, 2010; Flotho and Melchior, 2013). SUMO is also reversibly removed by the activity of SUMO isopeptidases, or SENPs (Mukhopadhyay and Dasso, 2007; Hickey *et al*., 2012). Although the dynamic cycling between conjugation and deconjugation can result in a relatively low steady-state level of a modified protein, SUMO modification nonetheless produces profound effects on substrate function in a variety of cellular pathways.

**Figure 1.**
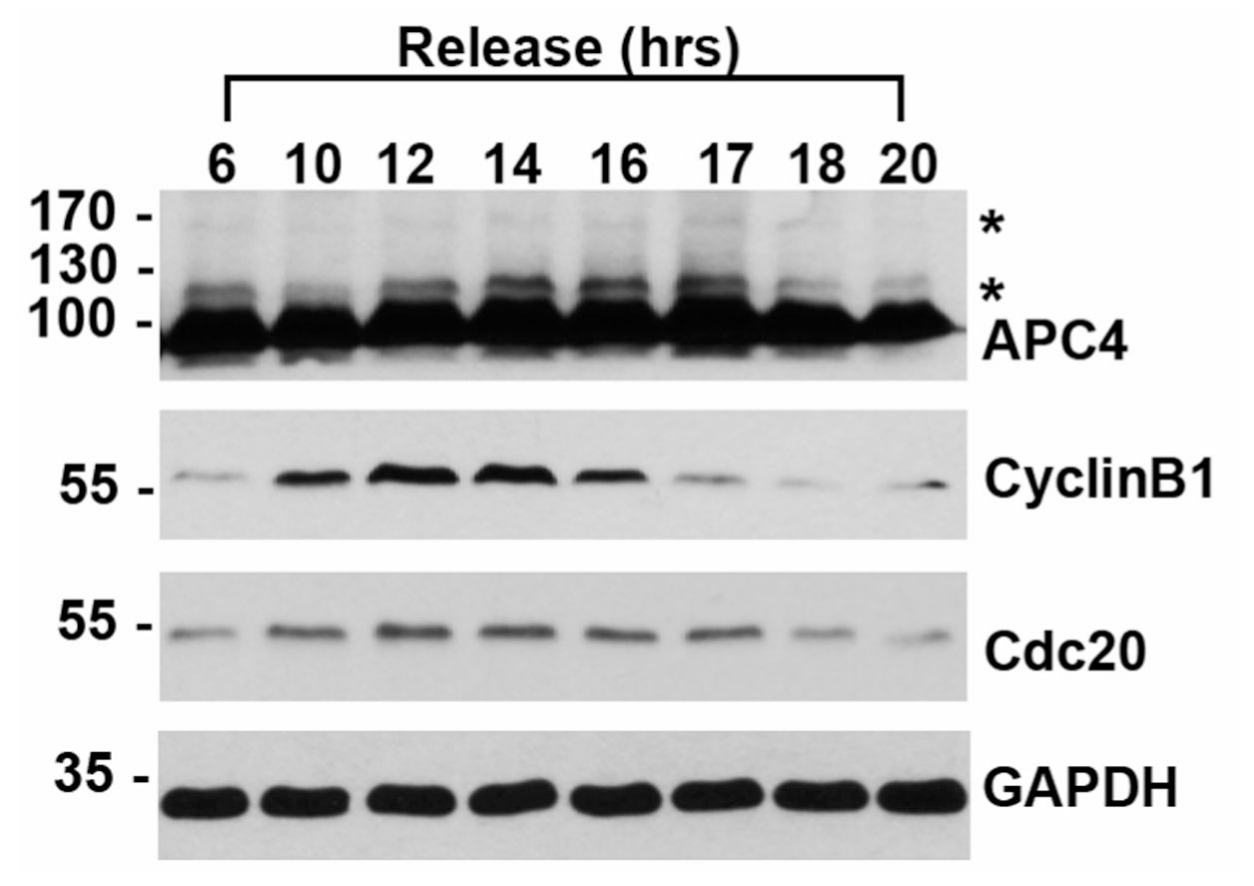
HeLa cell lysates were synchronized with a double thymidine block and released into mitosis, as indicated by immunoblots to CyclinB1 and Cdc20. Immunoblot to APC4 shows unmodified APC4 at ~100 kDa and SUMO-modified forms indicated by asterisks (*). Glyceraldehyde 3-phosphate dehydrogenase (GAPD) immunoblot is used as a loading control.

SUMO modification is most commonly detected by immunoblotting. Like other post-translational modifications such as ubiquitylation and PARylation, whole cell lysates immunoblotted for SUMO1 or SUMO2/3 reveal high molecular-weight smears due to the large number of cellular conjugates. Notably, SUMO modification of RanGAP1 can be identified as a band appearing on an immunoblot at ~90 kDa, an ~12 kDa shift above the unmodified 70 kDa protein (Matunis *et al*., 1996). To directly observe SUMO modification of specific substrates, immunoblots may also be performed with substrate-specific antibodies if modification levels are sufficiently high. For example, APC4 is a subunit of the Anaphase Promoting Complex/Cyclosome (APC/C) that is SUMO-modified robustly during the mitotic stage of the cell cycle as observed by immunoblot analysis of whole cell lysates from synchronized cells (Lee *et al*., 2018). Although SUMO modification by a single protein subunit can be detected as a ~12 kDa shift in molecular weight (Figure 1), this shift cannot be distinguished from modification by other ubiquitin-like proteins without further validation. An alternative method for demonstrating SUMO modification utilizes a cell line with His-SUMO, followed by Ni-NTA purification under denaturing conditions and immunoblotting for co-purifying proteins of interest (Lee *et al*., 2018). Complimentary approaches for detecting and validating SUMO-modified proteins utilize overexpression of SUMO E1 and E2 enzymes, or the depletion of SENPs, followed by immunoblotting for the protein of interest. Furthermore, recent advances in proteomic methods have made the sensitive identification of SUMO-modified proteins possible, leading to hundreds of SUMO substrates that must be further validated and characterized. (Matic *et al*., 2010; Schimmel *et al*., 2014; Cubeñas-Potts, *et al*., 2015).

In addition to these cellular assays, detection and verification of SUMO modification using biochemically purified components *in vitro* also represents an important approach to validating and characterizing novel SUMO substrates (Park-Sarge and Sarge, 2008; Werner *et al*., 2009; Yunus and Lima; 2009; Yang *et al*., 2018). With *in vitro* analysis of a substrate, mutants can also be particularly valuable in verifying specific modification sites. Here we describe a protocol for *in vitro* SUMO modification routinely used in our laboratory (Zhu *et al*., 2008; Reiter *et al*., 2016), as recently illustrated by our analysis of the APC4 subunit of APC/C (Lee *et al*., 2018).

### Materials and Reagents

1. Pipette tips (USA Scientific, catalog numbers: 1111-3700, 1111-0700, 1112-1720)
2. X-Ray Film, Blue Base for Autoradiography, 8 × 10 inch (RPI, Research Products International, catalog number: 248300)
3. Whatman™ Grade 2 Qualitative Filter Paper Sheet, Size 46 × 57 cm, Pore Size: 8 μm (GE Healthcare, Whatman™, catalog number: 1002-917)–cut into 10 × 9 cm rectangles
4. Clear plastic wrap (Fisher Scientific, catalog number: 22-305654)
5. 1.5 ml Eppendorf tube
6. Recombinant SUMO1 or SUMO2 (see Procedure B)
7. Recombinant SUMO E1 activating enzyme (Aos/Uba2) (see Procedure B)
8. Recombinant SUMO E2 conjugating enzyme (see Procedure B)
9. Appropriate plasmid containing substrate cDNA under control of T7 or SP6 promoters (For example, RanGAP1 can be used as a positive control, Plasmid) (Addgene, catalog number: 13379)
10. (Optional) Recombinant GST-RanGAP1 (Enzo Life Sciences, catalog number: BML-UW9755-0100)
11. TNT^®^ Quick Coupled Transcription/Translation System (Promega, catalog number: L1170 or L2080 for 40 reactions)
12. EasyTag™ L-[^35^S]-Methionine, 5 mCi (185 MBq), Stabilized Aqueous Solution (PerkinElmer, catalog number: NEG709A005MC)
13. Adenosine 5’-triphosphate (ATP) disodium salt hydrate (Sigma-Aldrich, catalog number: A1852)
14. Phosphocreatine disodium salt hydrate (Sigma-Aldrich, catalog number: P7936-10MG)
15. Pyrophosphatase, Inorganic from baker’s yeast (*S. cerevisiae*) (Sigma-Aldrich, catalog number: I1643)
16. HEPES (Molecular Biology Grade) (Fisher Scientific, Fisher BioReagents, catalog number: BP310-500)
17. Potassium hydroxide (ACS reagent, pellets) (Sigma-Aldrich, catalog number: 221473)
18. Potassium acetate (Molecular biology grade) (Sigma-Aldrich, catalog number: P1190)
19. Magnesium acetate tetrahydrate (Molecular biology grade) (Sigma-Aldrich, catalog number: M5661)
20. 1,4-Dithiothreitol (DTT) (Sigma-Aldrich, catalog number: 10197777001)
21. Acrylamide (Bio-Rad Laboratories, catalog number: 1610101)
22. 2% Bis Solution (Bis-acrylamide) (Bio-Rad Laboratories, catalog number: 1610142)
23. Ammonium persulfate (APS) (Bio-Rad Laboratories, catalog number: 1610700)
24. Sodium dodecyl sulfate (SDS) (Sigma-Aldrich, catalog number: 436143)
25. PageRuler™ Prestained Protein Ladder, 10 to 250 kDa (Thermo Fisher Scientific, catalog number: 26619)
26. Glycerol (Molecular biology grade) (Sigma-Aldrich, catalog number: G5516)
27. Bromophenol Blue (Sigma-Aldrich, catalog number: B0126)
28. β-mercaptoethanol (Sigma-Aldrich, catalog number: M3148)
29. Trizma^®^ base (Tris base) (Sigma-Aldrich, catalog number: T1503)
30. Hydrochloric acid (Molecular biology grade) (Sigma-Aldrich, catalog number: H1758)
31. Creatine phosphokinase (Sigma-Aldrich, catalog number: C3755)
32. Methanol (Fisher Scientific, catalog number: A412-4)
33. Acetic acid, glacial (Fisher Scientific, catalog number: A38-212)
34. Glycine (Sigma-Aldrich, catalog number: G7126-5KG)
35. SDS Sample Buffer (2x) (see Recipes)
36. SUMO Master Mix (see Recipes)
37. 12.5% SDS Polyacrylamide Gels (see Recipes, Note E)
38. Destaining solution (see Recipes)

### Equipment

1. Pipettes
2. Isotemp Digital Block Heater (Heats up to 100 °C) (Fisher Scientific, catalog number: 88-860-021)
3. Benchtop Centrifuge for 1.5 ml tubes (Eppendorf, model: 5430)
4. Small Stainless Steel Spatula (Fisher Scientific, catalog number: 21-401-10)
5. 30 °C water bath
6. Razor blade (Fisher Scientific, catalog number: 12-640)
7. Autoradiography cassette, 8 × 10 inches (RPI, Research Products International, catalog number: 420810)
8. Liquid Scintillation counter (Beckman Coulter, catalog number: 8043-30-1194)
9. Geiger counter
10. Radiation disposal receptacle (Provided by institution)
11. Mini-trans blot cell (Bio-Rad Laboratories)
12. Gel dryer (Bio-Rad Laboratories, model: 583)
13. Autoradiography case (VWR, catalog number: 95039-986)
14. Film processer
15. Benchtop Shaker (Thermo Fisher Scientific, catalog number: 88880022)

### Procedure

#### A. Safe handling of radioactive materials

Please note that this procedure utilizes radioactive ^[35]^S-methionine. All users must be appropriately trained to handle and dispose of radioactive materials. A dedicated laboratory space should also be demarcated for containment. Safe handling and disposal of all tips, tubes, and reagents are important for minimizing the risks of exposure contamination.

#### B. Preparation of SUMO enzymes from *E. coli*

Prior to performing the *in vitro* SUMO modification assay, recombinant SUMO and pathway enzymes must first be expressed in *E. coli* and purified. Methods for protein expression and purification have been described elsewhere (Yang *et al*., 2018).

#### C. *In vitro* transcription/translation of protein of interest

1. Remove a tube of rabbit reticulocyte from the kit stored at −80 °C and thaw on ice.
2. For each substrate, add 20 μl of rabbit reticulocyte to an Eppendorf tube on ice.
3. Return any remaining rabbit reticulocyte back to −80 °C for future use; avoid freeze/thaw cycles.
4. Add 2 μl of ^[35]^S-methionine to each tube, keep on ice.
5. Add 500 ng of plasmid DNA for substrate protein of interest to each reaction, keep on ice.
6. Incubate in a 30 °C water bath for at least 1 h (60-90 min).

#### D. *In vitro* SUMO modification

1. Each reaction will have 28 μl of SUMO Master Mix solution; place in a 1.5 ml Eppendorf tube, at room temperature. It is recommended to prepare fresh SUMO Master Mix solution (see Recipe 2).
2. Remove 2 μ;l of transcription/translation product and add to the 28 μl SUMO Master Mix, for a total volume of 30 μl, pipetting gently several times up and down to mix, at room temperature.
3. Incubate each reaction in a 30 °C water bath (see Note C).
4. Add 20 μl of sample buffer (2x) to stop the reaction.
5. Place on a 95 °C heat block for 5 min.
6. Briefly centrifuge at 10,000 × *g* for 30-60 sec, at room temperature.

#### E. SDS polyacrylamide gel electrophoresis

1. Prepare a 12.5% SDS-PAGE gel.
2. Load 10 μl of completed reaction to a well in a 12.5% SDS-PAGE gel, reserving a lane for 2 μl of protein ladder (lane 1).
3. Run SDS-PAGE at 70 V, room temperature, for approximately 20 min, or until the bottom dye indicator reaches just below the stacking gel and in the running gel. Run at 120 V, room temperature, for an additional 2 h. Stop electrophoresis before the 10 kDa molecular weight marker reaches the bottom of the gel (or until the bottom dye indicator just reaches the bottom of the gel).
4. Gently separate gel plates using a spatula. Gently slide surgical blade along sides of the glass plate to release gel and remove the stacking gel away from the running gel with blade and discard.
5. Carefully remove the gel and place in a small dish or plastic container (a pipet tip box lid is suitable) pre-filled with destaining buffer at room temperature.
6. Wash with gentle shaking for 10 min, at room temperature.
7. Carefully discard destaining buffer (free [^35^S]methionine may be in the solution, so pour in a radiation waste container).
8. Add MilliQ water and wash with gently shaking for 10 min, at room temperature. Repeat at least 3 times (see Notes).

#### F. Autoradiography

1. Lay a piece of Whatman™ paper on a clean benchtop space demarked as a radiation exposure area (room temperature).
2. Carefully place gel onto the Whatman™ paper so that lane 1 is the protein ladder.
3. Overlay the gel with a piece of plastic wrap.
4. Lay the gel and Whatman™ paper onto the gel dryer (the Whatman™ paper is placed directly onto the foam of the gel dryer and the plastic wrap is on top).
5. Gently cover the gel and dry for approximately 30 min (see Notes).
6. Carefully remove the plastic wrap from the gel and place on benchtop demarked as a radiation exposure area for 5 min.
7. In a dark room protected from light, place the dried gel affixed to the Whatman™ paper inside the cassette.
8. Carefully place a sheet of X-ray film on top of the gel.
9. Carefully close the cassette. Do not bring into contact with light.
10. Keep the cassette in a dark area overnight at room temperature.
11. Develop the exposed film according to manufacturer’s instructions.
  Note: For a video and detailed protocol for how to perform autoradiography, please refer to Karra et al. (2017).

### Data analysis

**Figure 2.**
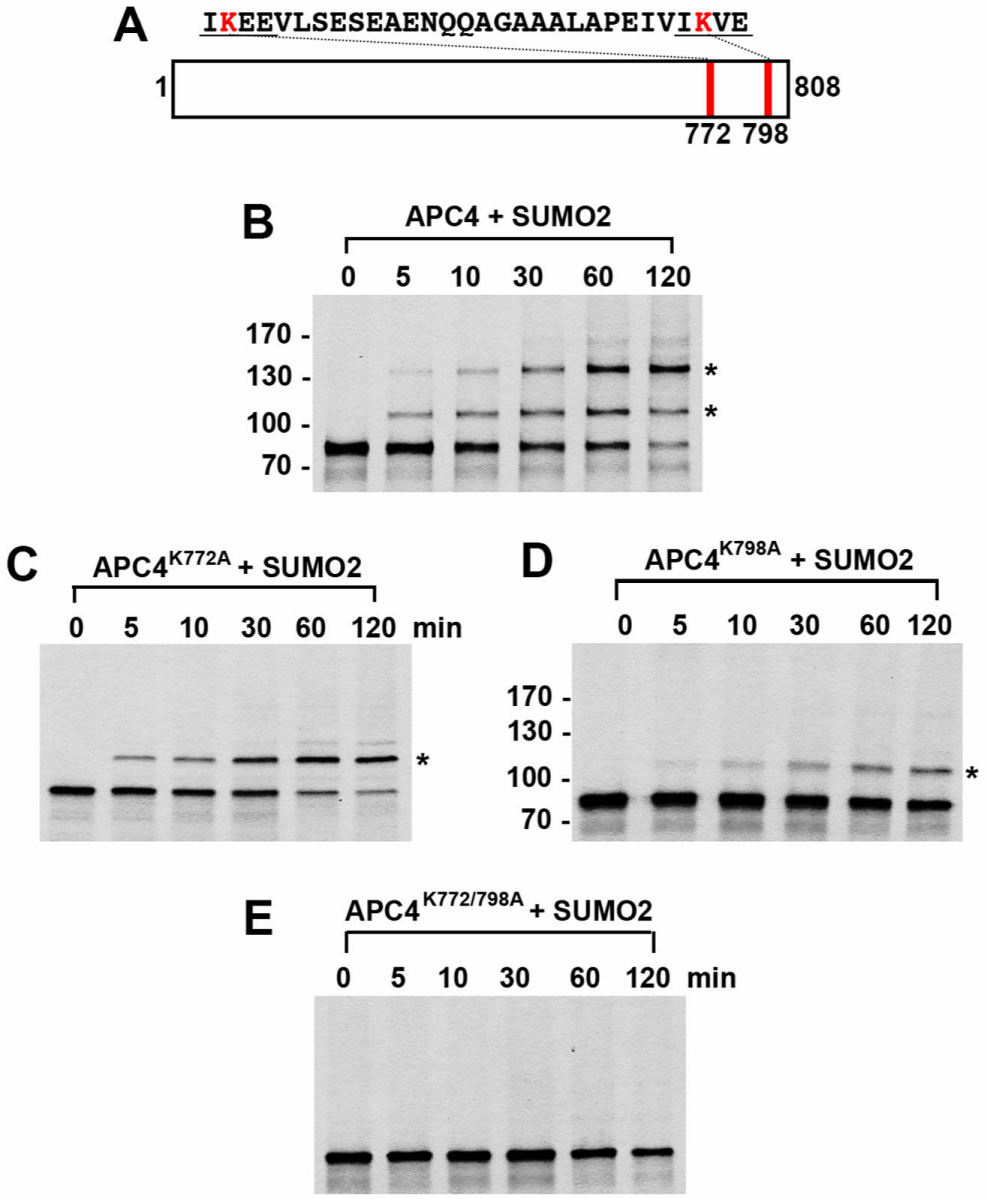
*In vitro* SUMO2 modification of APC4. APC4 is a 808 amino acid protein with two consensus SUMO sites at K772 and K798 (A). Full-length wild-type APC4 (B) or the indicated lysine to alanine substitution mutants (C, D, E) were expressed in rabbit reticulocyte lysate in the presence of [^35^S]-methionine and incubated for the indicated times in modification reactions containing SUMO E1 and E2 enzymes and SUMO2. Proteins were detected by SDS-PAGE and autoradiography. Asterisks indicate sumoylated forms of APC4. Please note that we have rearranged the panels of Figure 2.

1. Overlay developed film over dried gel and use molecular weight markers to demarcate molecular weights using a permanent pen.
2. Locate the unmodified protein of interest and any shifts in molecular weight (~12 kDa shift represents modification by a single SUMO, 24 kDa shift represents modification by two SUMOs, *etc*.) (Figure 2). It is possible for several SUMO consensus sites to be present in the protein of interest, and several SUMO-modified bands may be represented.
3. If amino acid substitutions have been made to SUMO consensus site lysines, high molecular weight band shifts should decrease in number until a complete SUMO mutant is generated (Figure 2).

### Notes

#### A. Identification of SUMO modification consensus sites and SUMO mutant generation

GPS SUMO is a web-based tool (Zhao *et al*, 2014) that identifies potential SUMO conjugation sites or SIM Motifs (Yunus and Lima, 2009). Input of the amino acid sequence in FASTA format is used.

#### B. Preparation of SUMO enzymes from *E. coli*

Depending on whether the protein of interest is modified by SUMO1 or SUMO2/3, the appropriate recombinant protein should be used in the assay. If unknown, it is recommended that modification by both SUMO1 and SUMO2/3 be tested. Of note, SUMO2/3 can generate polymeric chains due to a SUMO consensus site at K11 in both SUMO2 and SUMO3.

In human cells, the SUMO E1 enzyme is expressed as a heterodimer Aos1/Uba2. Co-expression and purification of these two proteins can be prepared in batch. The conjugating enzyme, Ubc9, is used in relatively high concentrations and may circumvent the requirement of an E3 ligating enzyme. Caution should be used, however, in possible misinterpretation of results and the possibility that E3’s may be critical for site-selective modification. It is also possible for some substrates to require an E3 ligase (such as the PIAS family of proteins [Zhu *et al*., 2008]) for efficient SUMO modification *in vitro*.

#### C. *In vitro* SUMO Modification–time-course analysis

In general, the time required for SUMO modification at 30 °C must be determined empirically, as the concentrations of SUMO enzymes in addition to increases in temperature (up to 37 °C) can change the efficiency of conjugation. Generally, with this protocol, SUMO substrates are modified within one hour. However, a time-course is recommended for each protein of interest, as some proteins are more readily SUMO-modified than others. Substrate recognition by SUMO conjugating enzymes or properties inherent in the SUMO protein itself can affect the kinetics of modification. For example, the presence of SUMO Interacting Motifs (SIMs) in the substrate protein can enhance SUMO modification. SIMs are composed of hydrophobic amino acids (V/1)-x-(V/1)-(V/1) flanked by an acidic residue. The hydrophobic amino acids in SIMs interact non-covalently with a hydrophobic pocket situated in the β_2_ strand on the surface of SUMO, creating a parallel or antiparallel β-strand conformation. SIM Motifs can also be identified through GPS SUMO (Zhao *et al*., 2014).

#### D. *In vitro* SUMO Modification–enzyme concentrations

The concentrations of enzymes used can also be empirically adjusted. For example, BLM and APC4 are readily SUMO-modified with the concentrations of SUMO enzymes described above, but RanGAP1 is more efficiently SUMO-modified and therefore requires a lower concentration of conjugation enzymes and shorter incubation periods to achieve complete modification. For example, 15 mM E1, 45 nM E2, and 0.5 μM SUMO proteins are required for complete SUMO modification of RanGAP1 within 5-10 min of incubation at 30 °C.

#### E. SDS polyacrylamide gel electrophoresis

It is critical to sufficiently wash the gel following destaining. The presence of destaining solution can cause the gel to crack during drying.

A 12.5% gel is recommended because free SUMO is a relatively small protein and this composition has proven to resolve proteins well in our experiments. A 4-15% gradient gel can also be used.

#### F. Autoradiography

Time for gel drying can vary. Monitor carefully to sufficiently dry the gel but taking care not to over-dry and generate cracks.

#### G. Positive and negative controls

As a positive control for SUMO modification, recombinant RanGAP1 can be used. RanGAP1 is a labile SUMO substrate and can be modified efficiently at room temperature. Concentrations of SUMO conjugating enzymes should be adjusted (15 mM E1, 45 nM E2, 0.5 μm SUMO1), as this protein is modified rapidly. Plasmids for expression in *E. coli* for protein purification (Werner *et al*., 2009) are available through Addgene (Plasmid #13379) or recombinant GST-RanGAP1 is commercially available (can be purchased from Enzo Life Sciences, Inc.) Lysine to arginine substitutions can be made as mutations on the substrate of interest to prevent SUMO conjugation. For example, one possible negative control is RanGAP1 with K526R cannot be modified by SUMO (Mahajan *et al*., 1998). Reactions without ATP or the SUMO E1 enzyme can also be utilized as negative controls for the reaction.

### Recipes

1. SDS Sample Buffer 2x
  0.313 M Tris-HCl, pH 6.8
  4% SDS
  20% glycerol
  3.5 M β-mercaptoethanol
  0.1% (w/v) bromophenol blue
  Store working aliquots at room temperature (and stock solutions at −20 °C)
2. SUMO Master Mix
  *Notes:*
  - The following enzymes and reagents should be generated in a 1.5 ml Eppendorf tube as a SUMO Master Mix.
  - Each reaction will have 28 μl of SUMO Master Mix + 2 μl of in vitro transcribed/translated protein of interest.
  - Prepare all solutions using ultrapure water (resistivity of 18.2 MΩ cm at 25 °C).
  - *As a negative control, a reaction without ATP can be used (SUMO conjugation is an ATP-dependent pathway). ATP should be added to the SUMO Master Mix last*.
    20 mM HEPES-KOH, pH 7.3
    110 mM potassium acetate
    2 mM magnesium acetate
    200 nM SUMO E1
    600 nM SUMO E2
    1 μM of SUMO1 or SUMO2
    20 units/ml creatine phosphokinase
    5 mM phosphocreatine
    0.6 units/ml inorganic pyrophosphatase
    1 mM DTT
    1 mM ATP
3. 12.5% SDS Polyacrylamide Gels
  Prepared as previously described (Guzzo *et al*., 2012):
  - Stacking gel acrylamide
    30% Acrylamide
    0.44% Bis-acrylamide
    Store at 4 °C protected from light
  - Stacking gel buffer
    0.5 M Tris-HCl, pH 6.8
    Store at 4 °C
  - Resolving gel acrylamide
    33.5% Acrylamide
    0.3% Bis-acrylamide
    Store at 4 °C protected from light
  - Resolving gel buffer
    1 M Tris-HCl, pH 9.1
    Store at 4 °C
  - Ammonium persulfate
    3% solution prepared in ultrapure water
    Store at −20 °C
  - SDS-PAGE running buffer (1x)
    0.025 M Tris-HCl
    0.192 M glycine
    0.1% SDS
    Prepare a working solution from a 4x stock by diluting 250 ml into 750 ml ultrapure waters
4. Destaining solution
  20% methanol, 10% acetic acid solution made in distilled/deionized water
